# Floristic diversity in different urban ecological niches of a southern European city

**DOI:** 10.1038/s41598-018-33346-6

**Published:** 2018-10-11

**Authors:** Mirko Salinitro, Alessandro Alessandrini, Alessandro Zappi, Dora Melucci, Annalisa Tassoni

**Affiliations:** 1 Department of Biological Geological and Environmental Sciences, University of Bologna, Via Irnerio 42, 40126 Bologna, Italy; 2 Institute for Cultural Heritage, Via Galliera 21, 40121 Bologna, Italy; 3 Department of Chemistry “Giacomo Ciamician”, University of Bologna, Via Selmi 2, 40126 Bologna, Italy

## Abstract

The present paper aimed at studying the vertical and horizontal spatial distribution, species richness and diversity of vascular plants in different urban ecological niches (urban habitats) by means of the case study of Bologna (Italy), a typical densely populated southern European city. A total of 477 species were found in the study area of the historical city centre, 30% of which were alien species. Alien plant species were mainly present among phanerophytes, while native plants were mainly therophytes and hemicryptophytes. The habitats that mostly contributed to the species total richness were semi-natural soils, followed by paved areas, walls, rooftops and manholes. The number of exclusive species decreased according to the selectiveness of the habitat, with manholes and rooftops being the most selective. The presence of hemicryptophytes constant decreased going from 27% of more humid habitats to 5% of more arid habitats, so that they can be considered a water availability biomarker. Urban habitat quality, measured by the number of native species, was directly proportional to the strength of selective factors and inversely proportional to the rate of disturbance, with roofs and semi-natural soils having, respectively, the highest and lowest quality. Finally, a relation between species richness and street characteristics, like width, orientation and type of flooring, was demonstrated.

## Introduction

Maintaining biodiversity and natural environments in urban areas, where more than half of the world’s population lives, is one of the biggest conservation challenges today^1,2^. The first key step to achieve this goal is to compile updated lists of *taxa* within modern cities^3,4^. Moreover, since cities are the major centres for the introduction and expansion of non-native species, these data sets can be a rich source of information on biological invasion and habitat homogenization^5–9^. According to Ives *et al*.^10^, cities are also hotspots of rare species, many of which have their distribution partially overlapping or even coinciding with large urban centres. Therefore, during the planning of rare species conservation strategies, urban territories should be incorporated into protection plans.

Generally, in the urban environment, it is possible to identify two distinct types of vegetation according to the growing substrate. For primary succession is meant the vegetation that occurs in cemented areas not in continuity with the soil (walls, roofs, gutters, etc.), where plants need special adaptations to withstand the very limiting conditions (e.g. lack of soil and water). With secondary succession is meant the vegetation in areas (cemented and not) in continuity with the natural soil (e.g. flowerbeds, road margins, sidewalks), where plant specialization is less important, as growing conditions are easier given the availability of substrate, water and nutrients (https://www.yumpu.com/it/document/view/32191011/aspetti-biologici-ed-ecologici-della-flora-infestante-enrico-avanzi). Urban soils of a secondary succession are typically composed of coarse stone matrices and building debris highly present in cemented areas. In general, they typically have higher pH values than natural soils, due to the presence of calcareous building materials or irrigation practices, and are discretely compacted^11,12^. In contrast, the soils of primary succession areas are *ex novo* formed by the accumulation of thin stone fragments, powders of different nature and organic matter. In the formation of these soils, a key role has been attributed to pioneering organisms such as mosses and lichens. On stony surfaces, mosses constitute an absorbent surface that retains dust, water and plant propagules, becoming a preferential germination site^13^.

**Figure 1:**
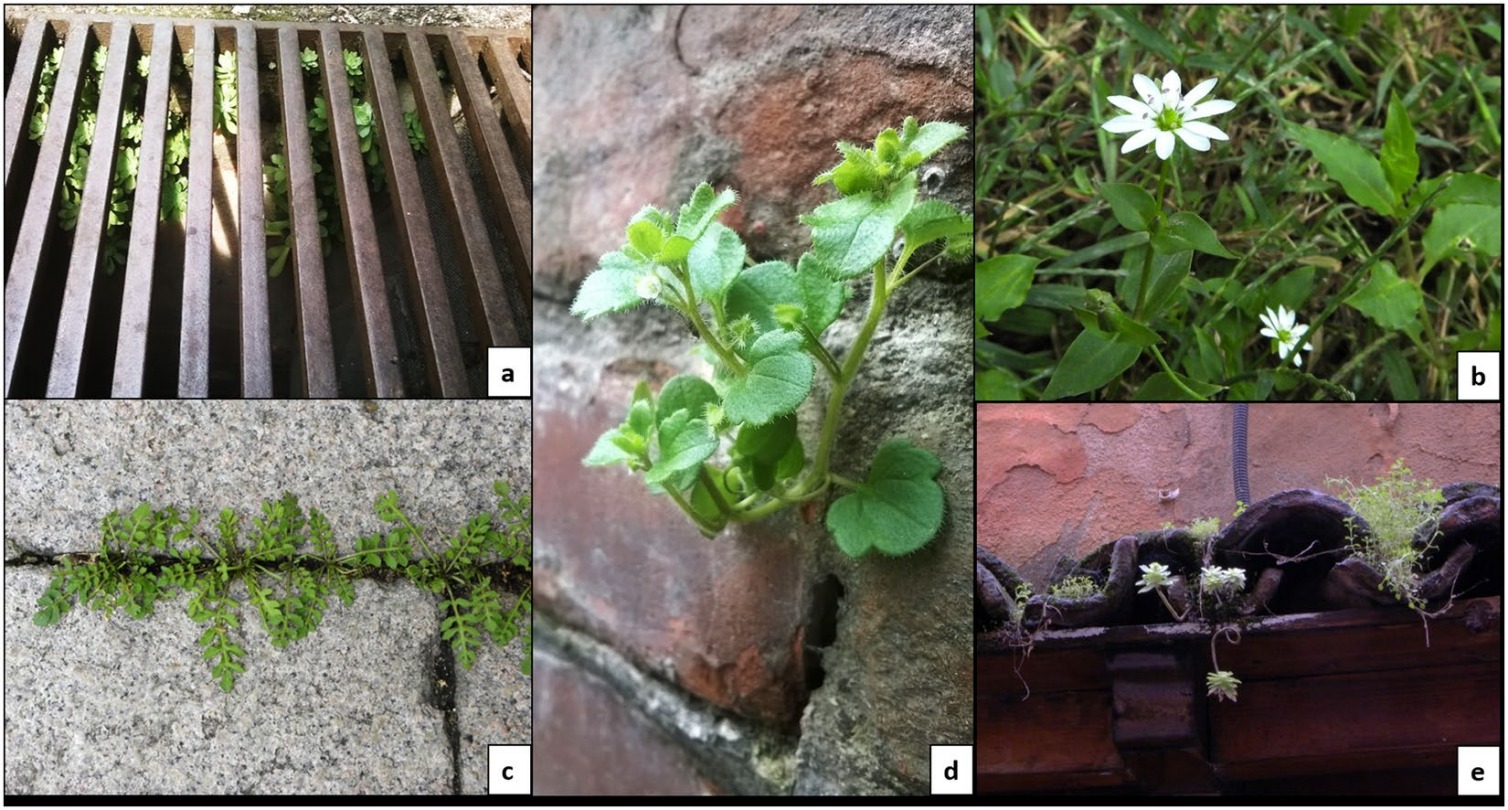
Examples of plants colonising the five urban ecological niches typical of a vertical succession in a southern European city. (**a**) *Sedum palmeri* S. Watson inside a manhole; (**b**) *Stellaria aquatica* (L.) Scop. in an over-irrigated meadow typical of semi-natural soils; (**c**) *Rorippa sylvestris* (L.) Besser on paving of trachyte plates; (**d**) *Veronica hederifolia* L. in an ancient wall habitat; (**e**) *Stellaria media* L. and *Sedum palmeri* S. Watson growing among tiles of a roof. Photographs made by M. Salinitro.

Urban plants occupy five main ecological niches (also referred as urban habitats throughout the manuscript) that can be vertically ordered according to their relative height from the ground (Fig. 1). This division was firstly introduced by Woodell^14^, who defined only two habitats, and later expanded by other authors^15^. In the present paper, the urban habitat division was further refined following the guidelines given by Fazio^16^. With increasing height, it is possible to find manholes and gratings, semi-natural soils, paved areas, walls, roofs and terraces (Fig. 1). Despite their proximity, these urban habitats have very diverse environmental parameters and host a different flora.

The lowest ecological niche is found below ground level and represented by manholes and gratings. The substrate that accumulates in these contexts is very rich in organic matter and moisture, while light intensity is very low. At ground level, semi-natural soils (such as irrigated meadows, flowerbeds, traffic dividers, etc.) are certainly the most heterogeneous habitats, as they are directly influenced by many agronomic practices. They range from compact soils, rich in coarse debris, to soft and humus-rich soils. The conditions of brightness are very heterogeneous, depending on the presence of trees, and, consequently, the grass cover can be abundant or totally absent. Moisture conditions are also variable, ranging from arid soils with no irrigations to over-irrigated soils that can host even marshy species. Another ground level niche is given by paved areas (such as pavements, roads, squares, etc.); in this case, the soil is spatially confined and, unlike semi-natural soils, temperatures are always quite high given the close contact with stone materials. These habitats exhibit strong drying conditions and soil compaction^12^ due to trampling. Vertical surfaces are represented by walls which can be distinguished in inhabited house walls, usually plastered and kept in good condition, and isolated walls, sometimes in direct contact with the ground as in the case of old fortifications and road embankments. The first type of walls generally hosts small plant species, which are usually regularly removed by maintenance, while the second category represents an ideal habitat for vegetation growth as the wall surface is degraded and maintenance is low or absent^13^. The highest located habitats in the city are roofs and terraces, which generally have tile coverage. On roofs the average temperature and brightness are particularly high, while water and substrate are very scarce.

The present research was aimed at describing plant species distribution, richness and diversity found in different urban ecological niches in relation to environmental parameters and levels of disturbance. It was investigated which urban habitat acts as *refugium* for native species, and which other represents a preferential site for alien species introduction. Moreover, the present paper aimed at pointing out the factors having a greater impact on floristic diversity in densely built historical urban cores, testing in particular the presence of green spaces and the relation with street width and building arrangement. During this survey, an inventory of the flora present within the historical centre of the city of Bologna was compiled, since no specific studies have been carried out over the last 120 years^17^.

## Methods

### Study area

The city of Bologna (Italy) is located in the southern part of the Po valley (latitude 44° 30′27″ 00 N, longitude 11° 21′5″ 04 E), close to Apennine mountains, between the Reno and Savena rivers. The altitude of the city centre varies from 75 m a.s.l. at Porta d’Azeglio to 46 m a.s.l. at the railway station with an average of 54 m a.s.l. (http://www.comune.bologna.it/media/files/f2_relazionegeologica.pdf).

Climatic data for the city are available through the Guglielmo Marconi Airport Weather Station database (https://weatherspark.com 2016). The warm season lasts from the beginning of June to the middle of September, with average minimum and maximum temperatures of 20 °C and 31 °C, respectively, while the cold season lasts from mid-November to early February with average minimum and maximum temperatures of 0 °C and 5 °C, respectively. Annual total precipitation amounts to approximately 700 mm, mostly concentrated in March-April and November. A period of semi-aridity can be detected in summer between July and August. Wind is generally weak, with average speed ranging from 0.2 to 5 m/s.

The soil below the city of Bologna is constituted by river sediments of different grain size, ranging from coarse gravel to clay. In the area formerly occupied by the Roman city (city centre), anthropic deposits form the most superficial layers of soil, whereas clay and anthropic deposits alternate in the rest of the historical centre^18^.

Bologna is crossed by two natural rivers (Rio Valle Scura and the Aposa stream) and several artificial canals. This dense water system is now completely buried underground and only a small portion of the Reno canal remains today, representing a peculiar wet habitat in the city centre.

The area considered in this study is the historical centre of Bologna enclosed by the avenues that follow the outline of the latest city wall circle of the XIV century. The selection of a limited sampling area allowed an in-depth and detailed survey of Bologna’s old city centre urban ecological niches, but, on the other hand, led to an underestimation of total urban plant richness as the areas outside the city walls, brownfields and railways of the city, were not considered in the experimental design.

Within the study area, the floristic data were collected by exploring all the environments in relation to the five types of habitat listed above, including abandoned areas, private gardens, public parks, ancient walls as well as roads, squares, houses and roofs.

### Data collection methods

The study was conducted between November 2014 and June 2016. For the correct location of the transepts and the choice of sampling areas, two preliminary surveys were conducted (November 2014 to January 2015 and April to June 2015), covering the entire historical urban area to identify the most significant places to be included in the study.

**Figure 2:**
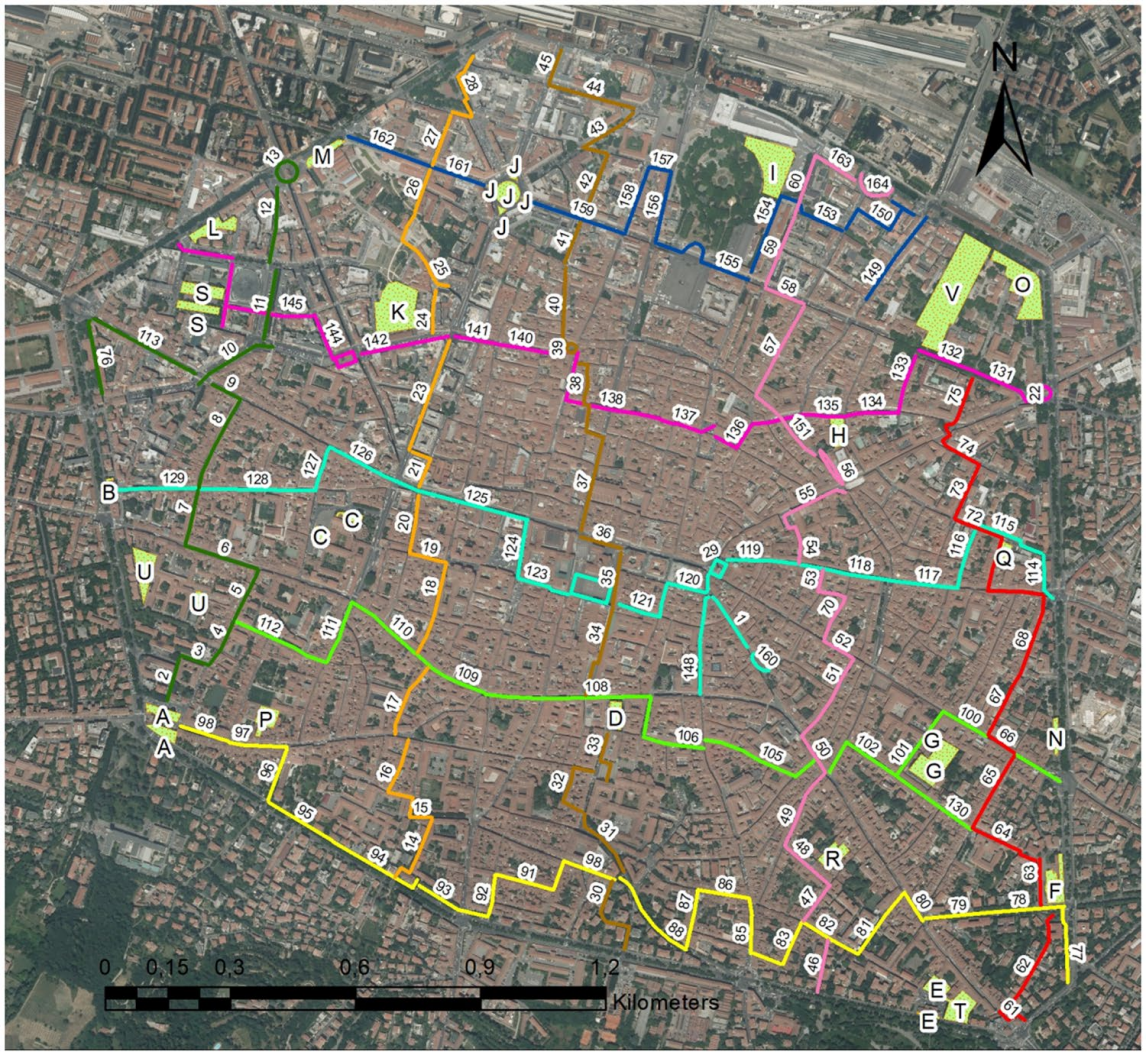
The study area of the historical city centre of Bologna. The coloured lines represent the 10 pre-determined transepts subdivided in 162 segments. Letters indicate the 22 green areas. Satellite image was downloaded from Google Earth (www.google.com/earth) and the graphical elaboration was made with the ArcMap 10.1 programme (ESRI, www.esri.com) by M. Salinitro.

Street width was measured by means of a laser distance meter and main orientation was assessed on a map. Ten transepts were thus defined and divided into 162 segments based on homogenous width and orientation of that portion of the street. The detection of the flora of Bologna was carried out along 10 predetermined transepts across the city centre (5 east-west and 5 north-south) and 22 public and private green areas (Fig. 2). For each segment, length, direction and width of the road, type of pavement, presence of adjacent green spaces or trees, growing species and growth habitat of each species were noted. In each segment, the plant species were determined by means of the presence-absence method, thus obtaining 162 small floristic lists associated with each segment. In addition, 22 green areas were also sampled, in many cases also including the ruins of the old city wall circle, and associated floristic lists were created. The floristic data related to the transepts and green areas were collected by means of 2 samplings (in addition to the preliminary surveys), one in autumn (September to November 2015) and one in spring (March to June 2016), to detect species with different vegetative periods.

During the sampling, all vascular plants that spontaneously occurred in each urban habitat were counted. A species was considered spontaneous when it was possible to ascertain its autonomous reproduction within the study area, either vegetatively or by seed. The seedlings of cultivated plants at least one year old were also counted^19^, as they indicated an environmental adaptation and a propensity for expansion of these species^20^.

The plant sampling and identification was carried out throughout the walkable area and, for inaccessible areas such as private courtyards, up to 5 meters away from the roadside. To better identify plants in inaccessible positions (e.g. roofs and terraces), binoculars were used. During the sampling, photographic material and herbarium specimens were collected to identify plants that were not immediately distinguishable. Identification of harvested species was carried out using identification keys^21^ or via the Acta Plantarum website (www.actaplantarum.org). The flora nomenclature was updated by means of the Acta Plantarum and The Plant List (www.theplantlist.org) websites. Chorotypes and life forms where inferred from Pignatti^21^ and the status of alien species from Galasso *et al*.^22^. The dispersal methods of species were assigned followed the guidelines of van der Pijl^23^ and Willson & Traveset^24^.

### Data processing

The sampled plant species were divided into 4 classes of rarity according to their frequency in the whole city centre. They were considered as *rare species* if found between 1 and 4 times, *scattered species* if found between 5 and 10 times, *common species* between 11 and 40 times, and *very common species* when found more than 40 times. The disturbance rate was assessed in each urban habitat taking into account the number of interventions per year (such as cutting, chemical weeding, uprooting, substrate removal, etc.) finalised at the elimination or reduction of natural vegetation. Only high intensity disturbance events, were considered. To determine the distribution of species related to the different urban habitats, a Non-metric Multi-Dimensional Scaling (NMDS) analysis was performed^25^. Computation was performed with the package *vegan*^26^ of the software *R* (R Core Team, Vienna, Austria). The dataset is a matrix of 5 rows (sampling habitats) and 477 columns (plant species). Bray distance was used for computation. Due to the low number of sampling habitats compared to the number of species, the NMDS algorithm did not reach a convergence, even after 1000 iterations, and the stress of the optimal solution was very low (<10^−5^).

Statistically significant difference between datasets regarding number of plant species in relation to street width, orientation and flooring type, was analysed by Microsoft Excel programme using one-way ANOVA test followed by post-hoc corrected two tail *t*-student test assuming equal variance (*p* < 0.05).

## Results and Discussion

### General overview of the flora of the historical city centre of Bologna

The present study was not aimed at describing the complete pattern of species richness in whole Bologna city area. The survey was only limited to the historical city centre without taking into consideration recent residential areas, industrial and constructional sites, brownfields or railway tracks located at the city outskirts even though these sites are often reported as having higher species richness than city centre locations and are also important for alien species introduction^8,27^.

The urban vascular flora of Bologna’s historical centre includes 477 identified species with 13 plants still uncertainly identified (only up to the level of family or genus), due to the non-optimal phenological phase for identification at the time of survey (Supplementary Table S1). However, it has been taken into account that constant removal of spontaneous vegetation or trampling make the sampling and identification of plants tricky. In addition, the city of Bologna hosts a large number of private courtyards, often inaccessible, that contain plant species that were impossible to sample, leading to an underestimation of the total urban species.

The number of species is in line with those of other similar medium-size Italian cities such as Ferrara (589 species)^28^, Cremona (390 species)^29^ and Rovigo (473)^30^ where, analogously to the present research, only the city centre was investigated.

In Bologna, the number of detected genres is 306, while that of botanical families is 101. The most represented plant families are in decreasing order: Asteraceae (52 species), Poaceae (45), Rosaceae (25), Fabaceae (25), Brassicaceae (22), which taken together represent more than the 35% of the total species. These data are in agreement with previous research^31^ that determined the most represented plant families in 26 urban floras around the world, confirming Asteraceae as the most frequent plants.

Overall, the percentage of alien species (30%) detected in the city of Bologna, mostly belonging to the Rosaceae and Asteraceae plant families (Supplementary Table S1), is lower in comparison to previous findings on European cities (average of 40%)^32^, probably due to the small dimension of Bologna’s sampling area (only urban city centre). Of all detected plants, 33% (159 species) are grown for ornamental purposes of which 84 are alien species that do not belong to the Italian flora. Of all alien plants, 43% originate from North and South America, 42% from Asia, 7% from Africa, 4% from other western and eastern European countries and 1% from Australia (Supplementary Table S1), as similarly shown for central European and Italian cities^33–36^. The American continent is the native place of most of the naturalized urban weeds (e.g. *Amaranthus* spp.), while the greatest part of ornamental species comes from Asia (e.g. *Ligustrum* spp.).

**Figure 3:**
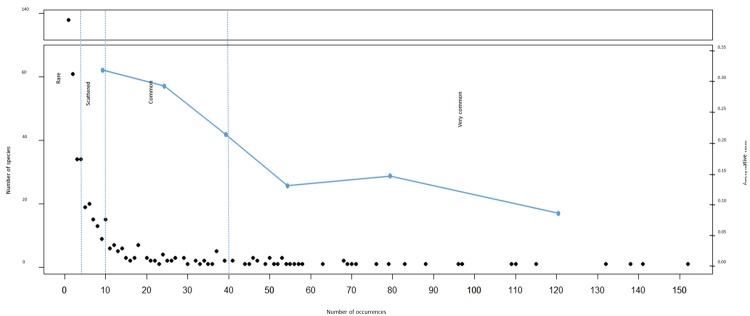
Trend of rarity categories of detected species. Black points represent the number of species belonging to a given rarity category, based on the number of occurrences of each species; blue line shows the ratio between the number of alien and native species.

Rare species (1 to 4 occurrences) constitute 56% of Bologna’s flora and 65% of detected alien species. These findings are in agreement with previous studies on other European cities^7–9^, such as Plzen (Czech Republic)^32^ and Berlin (Germany)^37^, and several cities in the north-east of Italy^33^. Most of the rare species cannot be considered naturalised yet, as it is demonstrated that infrequent species are liable to local extinctions^38^. These rare species, which in the urban environment are constantly compensated by the introduction of new alien species^39,40^, are the ones that highly contribute to increasing urban diversity^36^. Of the remaining detected plants, 19% are scattered species (5 to 10 occurrences), 16% common species (11 to 40 occurrences) and 9% very common species (more than 40 occurrences). As shown in Fig. 3, the alien/native ratio decreases from 0.32 for rare species to 0.10 for very common species. In fact, new alien species are usually present with ephemeral and localized populations. A similar pattern was previously shown for the city of Berlin^37^. The present results confirm the pattern known as “Law of infrequency” pointing out that in a floristic survey most of species are rare and few are common^41,42^.

The majority of species (91%) produced seeds within the city, while the remaining 9% was not found to bear seeds or fruits, implying that about 1/10 of the species was repeatedly introduced from outside the city or arrived by their own means as was also shown for several European cities by Ricotta *et al*.^36^. In fact, 47% of all detected species (Supplementary Table S1) uses wind as a primary means of seed dispersal (e.g. *Acer negundo* L.), 27% is introduced into the city by humans through cultivation and involuntary transport of seeds (e.g. *Malus domestica* Borkh. and *Solanum lycopersicum* L.), while the remaining part uses zoochorous seed dispersal (e.g. *Prunus avium* (L.) L.).

**Figure 4:**
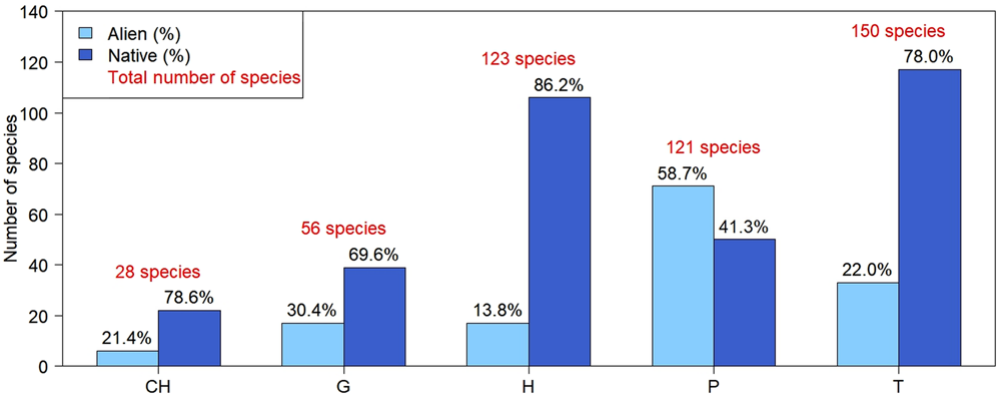
Abundance of different life forms in the current flora of the Bologna’s study area. CH: chamaephytes, G: geophytes, H: hemicryptophytes, P: phanerophytes, T: therophytes.

Not all species successfully colonise urban environments, as some adaptations are essential for their permanence and expansion. Life form analysis^41^ of the species found in Bologna showed that 31% are therophytes, 26% hemicryptophytes, 25% phanerophytes, 12% geophytes and 6% chamaephytes. Previous studies pointed out that therophytes are favoured within cities, as reported for central European cities^34^ and for Rome^43^. Moreover, due to the warmer urban environment, therophytes can accelerate their life cycles, producing more generations every year^44,45^, as observed for example for *Poa annua* L. The contribution of alien species to each category is variable, being high among phanerophytes (59%), chamaephytes (30%) and geophytes (30%), while poor among therophytes (22%) and hemicryptophytes (14%) (Fig. 4).

Chorotype analysis showed that the four most representative species categories are: naturalised invasive (15%), Euro-Mediterranean (18%), cultivated (14%) and paleotemperate (11%). These data are in agreement with those collected for other Italian cities like Trieste^33,46^, Cremona^29^ and Ferrara^28^, where invasive, Euro-Mediterranean and cosmopolites species are also most frequently present. Italian endemic species are totally absent within Bologna’s city historical centre.

It must be highlighted that Bologna, even if external to the distribution area of steno-Mediterranean plants, hosts twice the number of species than a coastal city like Trieste (6% against 2.6%)^46^. This high presence of steno-Mediterranean species could be ascribed to the Urban Heat Island (U.H.I) effect, which is basically a general increase in temperatures in urban areas due to a combined effect of anthropogenic emissions and the physical properties of building materials^47^. It was demonstrated that the U.H.I. is responsible for the increase of Bologna’s urban minimum temperatures of 3.5 °C with respect to the countryside^48^. This general increase of temperature could allow the growth of Mediterranean species (like *Pistacia terebinthus* Scop. and *Pinus pinea* L.).

### Urban ecological niches analysis and spatial patterns

Inside cities, ecological niches are spatially divided and vertically ordered. The spatial succession and the diverse environmental parameters are the source of the proper floristic diversity of each habitat. This study emphasizes and evaluates floristic identity of each urban habitat, and the above floristic survey was specifically designed for this purpose. Previous studies performed similar analyses focusing on phytosociological classes^49^. The habitat that mostly contributed to the total richness of the city was represented by semi-natural soils (such as parks and flowerbeds) with 426 species. Similar results were previously reported for Podgorica (Serbia)^45^ and Sheffield (UK)^50^ and more generally stated in an urban ecology mechanistic study^51^. Green areas give the largest contribution to urban biodiversity, both by enabling the growth of rare or nemoral species (e.g. in Bologna *Scilla bifolia* L. and *Carex divulsa* Stokes) and by being a preferential site for new introduced species. Paved areas (like roads and squares) and walls hosted, respectively, 191 and 155 species, while rooftops and manholes hosted, respectively, 31 and 21 species.

**Figure 5:**
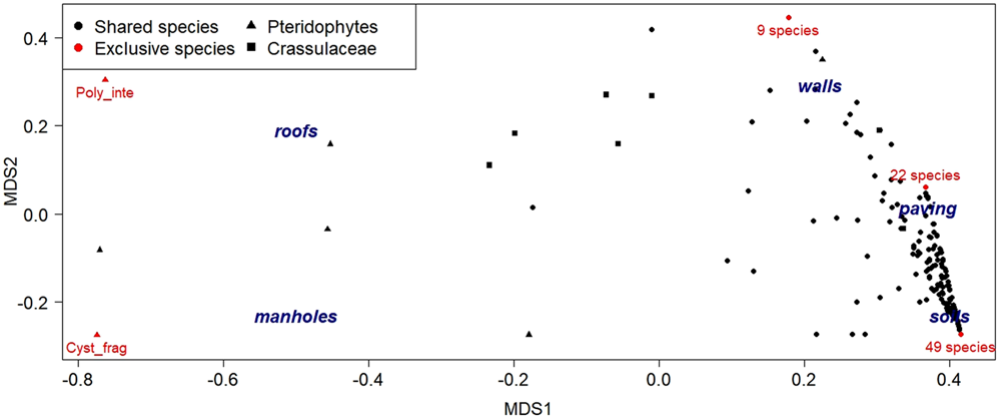
Non-metric Multi-Dimensional Scaling (NMDS) analysis of species distribution within the five sampled habitats. Red dots indicate the exclusive species of each habitat (total number or single species). Triangles and squares indicate, respectively, the grouped distributions of the two habitat-specific *taxa*, *Pteridophytes* and *Crassulaceae*. Poly_inte: *Polypodium interjectum*; Cyst_frag: *Cystopteris fragilis*.

The percentage of shared species between habitats ranged from 52% to 94%, with 4% of species being common to every habitat. The number of exclusive species decreased according to the selectiveness of the habitat, with 49, 22 and 9 exclusive species, respectively, in semi-natural soils, in paved areas and on walls (Fig. 5). Small shrubs, like *Clinopodium nepeta* (L.) Kuntze and *Capparis orientalis* Veill., were common on ancient walls, while paved habitats were rich in small plants resistant to trampling like *Sagina apetala* Ard. and *Polycarpon tetraphyllum* (L.) L. The most selective urban habitats were manholes and rooftops with only 1 exclusive species each. Two *taxa* can be identified as exclusive of specific habitats: Pteridophytes and Crassulaceae (Fig. 5). Pteridophytes such as *Asplenium scolopendrium* L. and *Cystopteris fragilis* (L.) Bernh. were mostly present in manholes and grids, while other fern species such as *Asplenium ceterach* L. and *Polypodium interjectum* Shivas were more typical of shadowy rooftops where the presence of porous bricks and tiles favoured their settlement. The Crassulaceae family was instead mainly distributed on roofs and walls (Fig. 5) with some species, like *Umbilicus rupestris* (Salisb.) Dandy and *Sedum spp*., preferentially found on sunny rooftops.

Given the vertical distribution of each habitat, specific environmental conditions (such as humidity and amount of soil) influence the life form patterns of their exclusive and not exclusive plants species. Under extremely arid conditions, like roofs, therophytes are largely dominant (90%). Phanerophytes and chamaephytes are abundant on ancient walls (43% and 6%, respectively), while geophytes are mostly present in semi-natural soils (13%). Hemicryptophytes showed a constant decrease from humid habitats, like manholes and soils (27%), to paving (24%), walls (16%) and roofs (5%) so that they can be considered as a biomarker for water availability.

Habitat quality is based on the presence of native species: the higher the number of native species, the higher the quality of the habitat itself^11^. In the present study, the number of native species detected in each habitat was directly proportional to the strength of selective factors (e.g. extreme temperatures, lack of soil, drought, etc.) and inversely proportional to the rate of disturbance. The urban habitats with the highest quality were roofs (94% of native species) while lowest quality was detected in semi-natural soils (59%). The results agreed both with disturbance rate which is decennial for roofs and three or more times a year in semi-natural soils, and strength of selective factors. Collected data may lead for the first time to a correlation between plant life forms and habitat quality. In fact, more selective habitats, having a high number of therophytes and hemicryptophytes, were also richer in native species, while less selective habitats, with numerous woody species, were richer in alien species.

**Figure 6:**
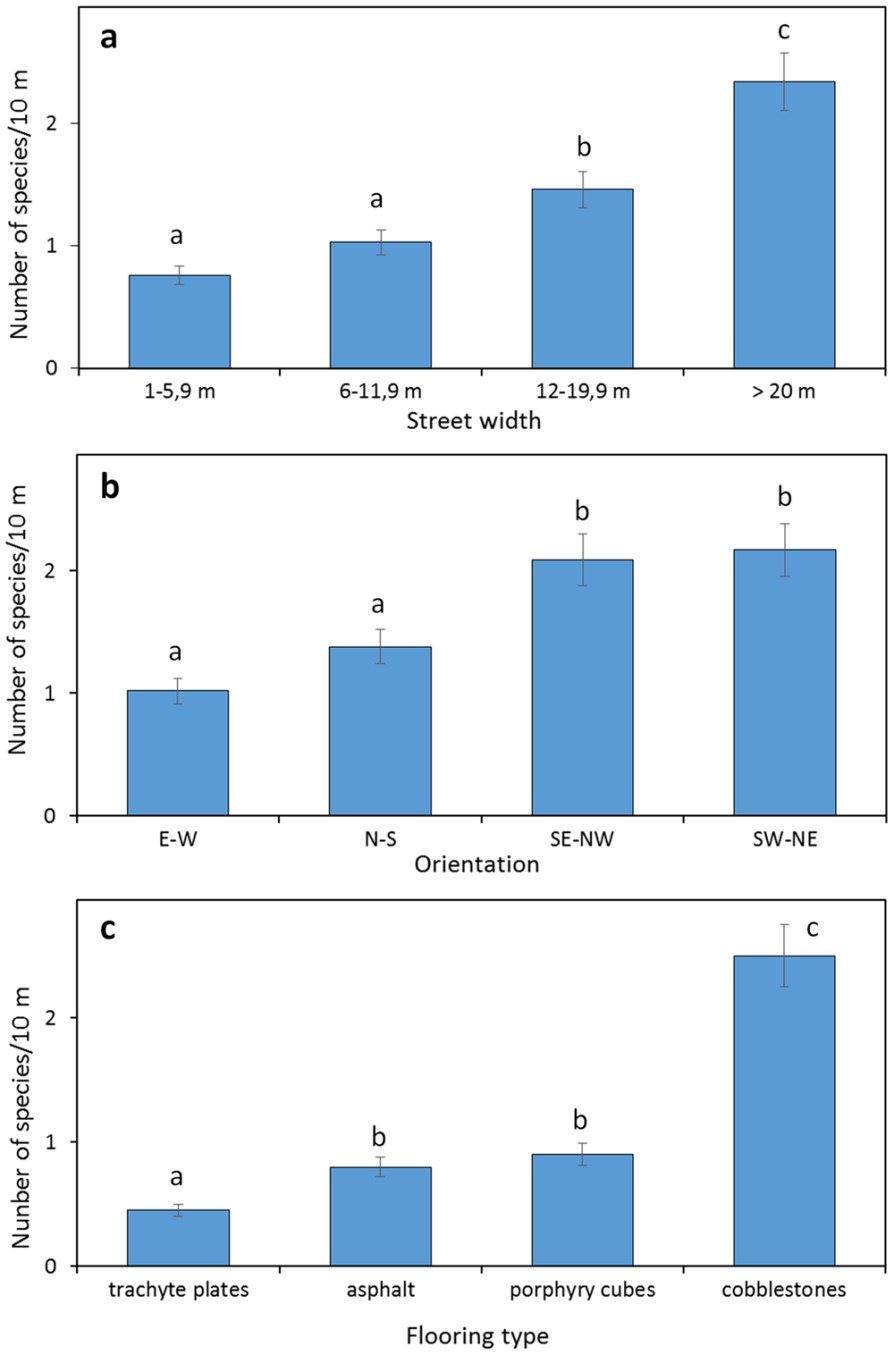
Relation between species richness and street characteristics. (**a**) Street width; (**b**) street orientation; (**c**) flooring type. Data show the number of plant species per 10 meters ± SD. Different letters indicate statistically significant differences between average data calculated by one way ANOVA test followed by post-hoc corrected two tail *t*-student test assuming equal variance (*p* < 0.05).

A direct proportional relation was also detected between species richness and street width (Fig. 6a). Large streets and squares wider than 20 m hosted an average of 2.3 species/10 m. Generally, species richness decreased proportionally to street width to reach 0.8 species/10 m in streets less than 6 m wide. This phenomenon is not a simple “area effect”. In fact, in streets of a densely populated city, plants do not grow in the whole area available, but only at the edges. Road vegetation can therefore be considered as linear and street width seems consequently not involved in increasing the space available for plant growth. On the other hand, street width is very important in determining insolation, temperature and light amounts, making wider streets more suitable for the growth of plant species. At the same time, the orientation of the sampling segments also had an effect. Data pointed out that E-W and N-S oriented streets showed the smallest number of species (1.0 and 1.4 species/10 m, respectively). In contrast, the number of species was significantly higher (2.1 species/10 m) in segments with intermediate orientations SE-NW and SW-NE (Fig. 6b). Having partial insolation on both sides of the street for a few hours per day, these segments create a more suitable environment for plant growth with respect to E-W or N-S orientated segments. In fact, one side of E-W streets always remains in total shade while the other is exposed to strong insolation. In N-S streets, on the other hand, sunlight can reach the ground only for few hours per day.

The flooring type seemed also to influence the settlement and the survival possibilities of urban plants. The detected differences ranged among 0.5 species/10 m for trachyte plates, 0.8 species/10 m for asphalt and 0.9 species/10 m for porphyry cubes (Fig. 6c). Very different was the situation of cobblestone pavings, which hosted on average 2.5 species/10 m, 5 to 3-fold higher with respect to other flooring types. Cobblestones create a very heterogeneous surface that allows the accumulation of organic matter and prevents the crushing of young seedlings in the early stages of life. In addition, the permeability of the flooring allows the soil to be soaked during rains, making this paving richer in species.

According to our knowledge, no other study has been reported regarding the relation between species richness and street characteristics like width, orientation and type of flooring, making the above-mentioned considerations totally innovative.

**Figure 7:**
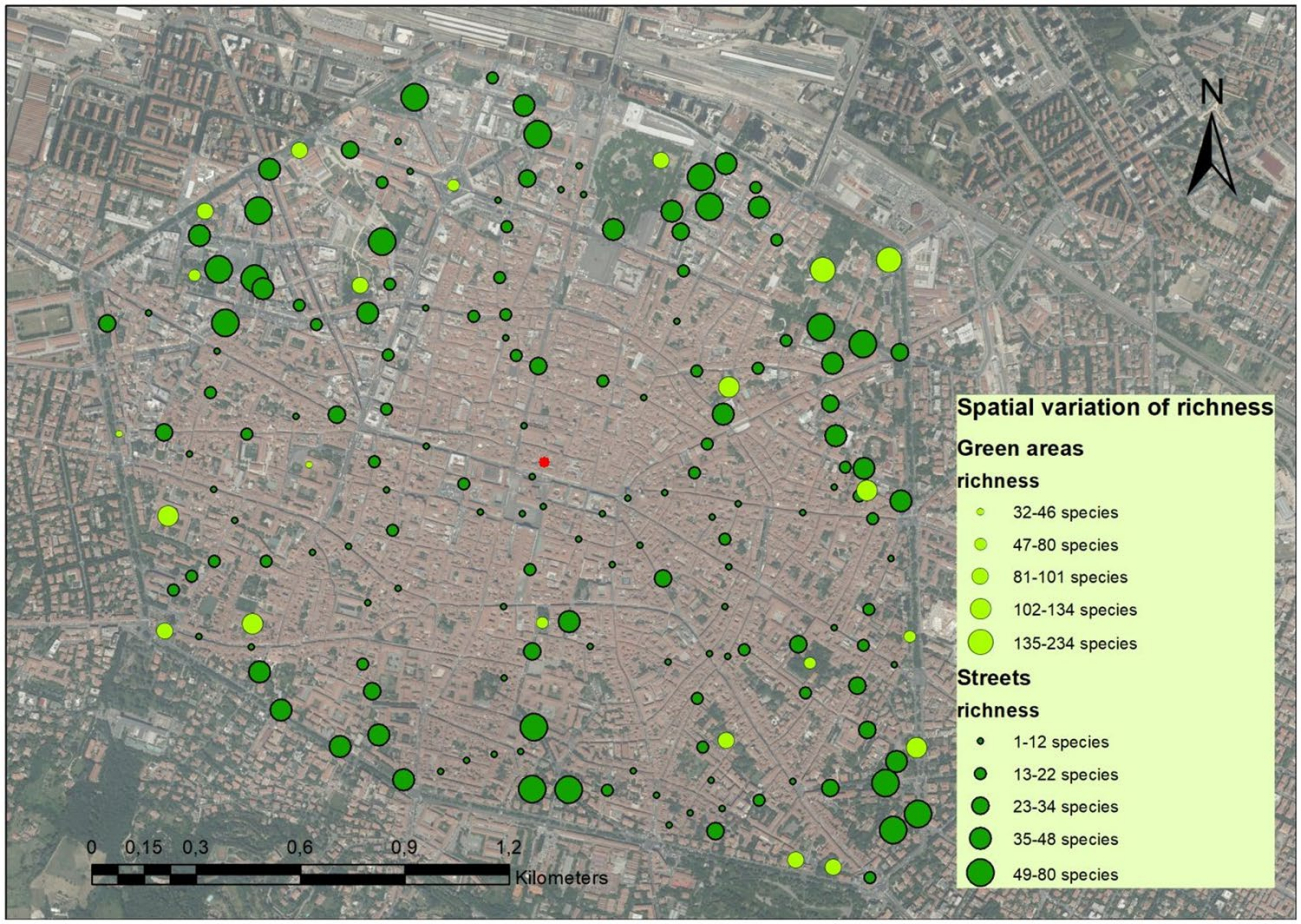
Spatial variation of species richness in streets (dark green dots) and green areas (light green dots) of the Bologna city centre. Satellite image was downloaded from Google Earth (www.google.com/earth) and the graphical elaboration was made with the ArcMap 10.1 programme (ESRI, www.esri.com) by M. Salinitro. The red dot represents the centroid of the study area as calculated by ArcMap programme.

Plant distribution evidenced that the total number of species generally increased from the city centre to the outer part of the study area (Fig. 7). In particular, the sampled segments contained on average 15 species within a radius of 500 meters from the centre, 30 species between 500 m and 1 km from the centre, and 46 species between 1 and 1.5 km from the centre. This observation could be explained by the progressive decrease in the number of impervious surfaces (such as buildings and roads) and by the increase in gardens, which was demonstrated to be the primary factor influencing plant richness^52^. Moreover, the outer parts of the study area could be more exposed to the introduction of species (via seed transportation) coming from peripheral parts of the city and playing an important role in increasing species richness. Analogously, green areas showed a similar pattern of species richness distribution (Fig. 7). Similar data were previously reported in a specific study on Paris (France) urban lawns^52^.

Finally, many protected species were found during the survey, indicating a shelter function of some urban habitats, in particular of semi-natural soil. Among the protected and endangered plants, *Asplenium scolopendrium* L., *Euphorbia hirsuta* L. and *Galanthus nivalis* L. were detected. Moreover, the alien species *Cotoneaster hissaricus* Pojark. was recorded for the first time in Italy, on ancient city walls.

## Conclusions

The present study showed that, in the urban environment of Bologna, it was possible to identify a spatial distribution of plant species richness among different habitats in a vertical succession. Manholes and rooftops were found to be the most selective habitats, whereas semi-natural soils were least selective. The species richness was primarily influenced by the presence of semi-natural soils, including green areas. Some of the investigated habitats showed to be of a great importance as *refugium* for native species. Interestingly, the most relevant in this conservation role are roofs, which are also those most subjected to human maintenance interventions.

Thanks to the diversity of habitats, Bologna’s urban landscape showed to have a high species diversity, even including rare and endangered species^53^, pointing out the urgent need for nature protection and conservation as many of the urban habitats are in fact unique^38^. Gardens and parks constitute the major source of propagules of alien species, as a great number of new alien species were introduced through voluntary cultivation, reflecting garden trends and fashion of the last decades^54^. In addition, the present study highlighted for the first time that the structure of the city (street width, orientation and flooring type) could be of primary importance in plant species distribution and richness within an urban environment.

Finally, even though the city of Bologna did not host endemisms, many rare and protected entities were detected, such as *Orchis purpurea* Huds., *Cephalanthera damasonium* (Mill.) Druce and *Asplenium scolopendrium*. To allow species to thrive in urban areas, management of green spaces and weed control in impervious areas should take into account the biology and phenology of these endangered species.

## Electronic supplementary material

Supplementary Table S1

## Data Availability

All the data supporting results presented here have been provided within the manuscript’s Figures, Tables and Supplementary Material. All data generated or analysed during this study are included in this published article (and its Supplementary Information files).
