# Supplementary material for: Floristic diversity in different urban ecological niches of a southern European city
Source: Sci Rep. 2018 Oct 11;8:15110. doi: 10.1038/s41598-018-33346-6 (PMC6181971; doi:10.1038/s41598-018-33346-6)
Supplement: Supplementary file 1 — Supplementary Table S1 [file 41598_2018_33346_MOESM1_ESM.pdf]

Title: Floristic diversity in different urban ecological niches of a southern European city

Authors: Mirko Salinitro[1], Alessandro Alessandrini[2], Alessandro Zappi[3], Dora Melucci[3], Annalisa Tassoni[1]*

[1]Department of Biological Geological and Environmental Sciences; University of Bologna, Via Irnerio 42, 40126, Bologna, Italy
[2]Institute for Cultural Heritage, Via Galliera 21, 40121, Bologna, Italy
[3]Department of Chemistry "Giacomo Ciamician", University of Bologna, Via Selmi 2, 40126, Bologna, Italy

*Corresponding author: AT, Department of Biological Geological and Environmental Sciences, Via Irnerio 42, 40126, Bologna, Italy; e-mail: annalisa.tassoni2@unibo.it

Supplementary Table S1. List of plant species found in the historical centre of the city of Bologna (Italy).
Cf.: indicates an uncertainly identified species followed by the name of the most probable identification. T: therophytes, H: hemicryptophytes, P: phanerophytes, G: geophytes, CH: chamaephytes, NP: nano-phanerophytes. S: semi-natural soils, P: paved areas, W: walls, R: roofs, M: manholes. Culton: a plant that derives from cultivation (e.g. cultivars) as proposed by Hetterscheid & Brandenburg (1985)[1]. Chorotypes and life forms are reported as in Pignatti (1982)[2]; casual and naturalised species as in Galasso et al. (2018)[3]. When in brackets the letters indicate a secondary habitat. N.D.= not determined.

Species	Family	Life form	Alien	Origin	Habitats	Cultivated	Status
Acalypha australis L.	Euphorbiaceae	T	✓	Asia	S		Naturalised
Acanthus mollis L.	Acanthaceae	H			S	x	
Acer campestre L.	Aceraceae	P			S (W)	x	
Acer negundo L.	Aceraceae	P	✓	North America	S (P)	x	Naturalised
Acer pseudoplatanus L.	Aceraceae	P			S	x	
Acer saccharinum L.	Aceraceae	P	✓	North America	S	x	Casual
Achillea roseoalba Ehrend.	Asteraceae	H			S		
Adiantum capillus-veneris L.	Adiantaceae	G			W	x	
Aegopodium podagraria L.	Apiaceae	G			S		
Aesculus hippocastanum L.	Hippocastanaceae	P	✓	East Europe	S	x	Naturalised
Ailanthus altissima (Mill.) Swingle	Simaroubaceae	P	✓	East Asia	S P (W)		Naturalised
Ajuga reptans L.	Lamiaceae	CH			S		
Albizia julibrissin Durazz.	Fabaceae	P	✓	East Asia	S	x	Casual
Alcea rosea L.	Malvaceae	H	✓	East Asia	S	x	Naturalised
Alliaria petiolata (M. Bieb.) Cavara & Grande	Brassicaceae	H			S		
Allium ampeloprasum L.	Liliaceae	G			S	x	
Allium roseum L.	Amaryllidaceae	G			S		
Allium triquetrum L.	Liliaceae	G			S	x	
Allium vineale L.	Liliaceae	G			S (W)		
Alopecurus myosuroides Huds.	Poaceae	T			W (S) (R)		
Amaranthus blitoides S. Watson	Amaranthaceae	T	✓	North America	S P		Naturalised
Amaranthus blitum L.	Amaranthaceae	T			S P		
Amaranthus deflexus L.	Amaranthaceae	T	✓	North America	S P		Naturalised

Amaranthus retroflexus L.	Amaranthaceae	T	✓	North America	S P		Naturalised
Amaranthus tuberculatus (Moq. ex DC.) J.D. Sauer	Amaranthaceae	T	✓	North America	S		Naturalised
Ammi majus L.	Apiaceae	T			S		
Anagallis arvensis L.	Primulaceae	T			S		
Anisantha diandra (Roth) Tzvelev	Poaceae	T			S		
Anisantha rubens (L.) Nevski	Poaceae	T			W R		
Anisantha sterilis (L.) Nevski	Poaceae	T			S (P)		
Anthoxanthum odoratum L.	Poaceae	H			S		
Anthriscus sylvestris (L.) Hoffm.	Apiaceae	H			S P		
Antirrhinum majus L.	Scrophulariaceae	CH	✓	Culton	W (P)	x	Naturalised
Apium graveolens L.	Apiaceae	H			S	x	
Aquilegia spp.	Ranunculaceae	H		Culton	S (P)	x	
Arabidopsis thaliana (L.) Heynh.	Brassicaceae	T			S P (W)		
Arctium minus (Hill) Bernh.	Asteraceae	H			S		
Arenaria serpyllifolia L. subsp. *serpyllifolia*	Caryophyllaceae	T			P W		
Arenaria serpyllifolia subsp. *leptoclados* (Rchb.) Nyman.	Caryophyllaceae	T			W (P)		
Artemisia verlotiorum Lamotte	Asteraceae	H	✓	East Asia	S		Naturalised
Artemisia vulgaris L.	Asteraceae	H			S		
Arum italicum Mill.	Araceae	G			S		
Arundo donax L.	Poaceae	G			S		
Asparagus acutifolius L.	Asparagaceae	NP			S		
Asparagus aethiopicus L.	Asparagaceae	G	✓	Africa	S	x	Casual
Aspidistra elatior Blume	Liliaceae	G	✓	Asia	S	x	Casual
Asplenium adiantum-nigrum L.	Aspleniaceae	H			W M		
Asplenium ceterach L.	Aspleniaceae	H			R M		
Asplenium scolopendrium L.	Aspleniaceae	H			W M		
Asplenium trichomanes L.	Aspleniaceae	H			W (R) (M)		
Atriplex patula (L.) Aellen	Chenopodiaceae	T			S		
Avena barbata Pott ex Link	Poaceae	T			S		

Avena sterilis L.	Poaceae	T			S (P)		
Ballota nigra L.	Lamiaceae	H			S W		
Bellis perennis L.	Asteraceae	H			S (P)		
Beta vulgaris L.	Amaranthaceae	H			S		
Brachypodium sylvaticum (Huds.) P. Beauv.	Poaceae	H			S	x	
Bromus hordeaceus L.	Poaceae	T			S		
Broussonetia papyrifera (L.) Vent.	Moraceae	P	✓	East Asia	S (P)		Naturalised
Buddleja davidii Franch.	Scrophulariaceae	P	✓	East Asia	S	x	Naturalised
Buglossoides purpurocaerulea (L.) I.M. Johnst.	Boraginaceae	H			S	x	
Buxus sempervirens L.	Buxaceae	NP			S (W)	x	
Calendula officinalis L.	Asteraceae	T			S	x	
Calepina irregularis (Asso) Thell.	Brassicaceae	T			S		
Calystegia sepium (L.) R. Br.	Convolvulaceae	H			S		
Campsis radicans (L.) Seem. ex Bureau	Bignoniaceae	P	✓	North America	S (P) (W)	x	Casual
Cannabis indica L.	Cannabaceae	T	✓	Asia	S		Casual
Capparis orientalis Veill.	Capparaceae	NP			W		
Capsella bursa-pastoris (L.) Medik.	Brassicaceae	H			S P		
Capsella rubella Reut.	Brassicaceae	T			P (S)		
Capsicum annuum L.	Solanaceae	T	✓	South America	S	x	Casual
Cardamine hirsuta L.	Brassicaceae	T			S P W R		
Cardaria draba (L.) Desv.	Brassicaceae	G			S		
Carduus pycnocephalus L.	Asteraceae	H			S P		
Carex divulsa Stokes	Cyperaceae	H			S (P)		
Carex pendula Huds.	Cyperaceae	H			S		
Carpinus betulus L.	Betulaceae	P			S	x	
Catapodium rigidum (L.) C.E. Hubb.	Poaceae	T			W (S)	x	
Celtis australis L.	Ulmaceae	P			S P W	x	
Celtis occidentalis L.	Ulmaceae	P	✓	North America	S	x	Naturalised
Centaurea nigrescens Willd.	Asteraceae	H			S		
Centranthus ruber (L.) DC.	Caprifoliaceae	CH			W	x	
Cephalanthera damasonium (Mill.) Druce	Orchidaceae	G			S		

Cephalotaxus harringtonii (Knight ex J. Forbes) K. Koch	Cepahlotaxaceae	P	✓	South America	S	x	Casual
Cerastium brachypetalum Desp. ex Pers.	Caryophyllaceae	T			S		
Cerastium glomeratum Thuill.	Caryophyllaceae	T			S P W R		
Ceratochloa cathartica (Vahl) Herter	Poaceae	H	✓	South America	S		
Cercis siliquastrum L.	Fabaceae	P			S (P) (W)	x	Naturalised
Chelidonium majus L.	Papaveraceae	H			S P W		
Chenopodium album L.	Chenopodiaceae	T			S P (W)		
Chenopodium polyspermum L.	Chenopodiaceae	T			S		
Chimonanthus praecox (L.) Link	Calycanthaceae	P		North America	S	x	
Chlorophytum comosum (Thunb.) Jacques	Asparagaceae	H	✓	South Africa	S P	x	Casual
Chondrilla juncea L.	Asteraceae	H			S P		
Cichorium intybus L.	Asteraceae	H			S		
Cirsium arvense (L.) Scop.	Asteraceae	G			S (P)		
Cirsium vulgare (Savi) Ten.	Asteraceae	H			S P		
Claytonia perfoliata Donn ex Willd.	Portulacaceae	T	✓	North America	S		Naturalised
Clematis vitalba L.	Ranunculaceae	P			S		
Clerodendrum trichotomum Thunb.	Verbenaceae	P	✓	East Asia	S P	x	Naturalised
Clinopodium nepeta (L.) Kuntze	Lamiaceae	H			S P W		
Commelina communis L.	Commelinaceae	G	✓	East Asia	S (P) (M)	x	Naturalised
Conium maculatum L.	Apiaceae	H			S		
Convolvulus arvensis L.	Convolvulaceae	G			S (P)		
Cornus sanguinea L.	Cornaceae	P			S	x	
Corylus avellana L.	Fagaceae	P			S	x	
Cotoneaster hissaricus Pojark	Rosaceae	NP	✓	North America	W		Naturalised
Cotoneaster horizontalis Decne.	Rosaceae	NP	✓	East Asia	S W	x	Naturalised
Crambe hispanica L.	Brassicaceae	T			P		
Crataegus monogyna Jacq.	Rosaceae	P			S W		
Crepis capillaris (L.) Wallr.	Asteraceae	T			S		
Crepis foetida L.	Asteraceae	T			W (P)		
Crepis pulchra L.	Asteraceae	T			S		

Crepis rhoeadifolia M. Bieb.	Asteraceae	T			S		
Crepis sancta subsp. *nemausensis* (P.Fourn.) Babc.	Asteraceae	T			S P (W)		
Crepis vesicaria L.	Asteraceae	H			S (P)		
Cupressus sempervirens L.	Cupressaceae	P	✓	North Africa	W	x	Naturalised
Cuscuta campestris Yunck.	Convolvulaceae	T	✓	North America	S		Naturalised
Cyclamen hederifolium Aiton	Primulaceae	G			S	x	
Cymbalaria muralis G. Gaertn., B. Mey., & Scherb.	Scrophulariaceae	T			P W (S) (M)		
Cynodon dactylon (L.) Pers.	Poaceae	G			S P		
Cyperus eragrostis Lam.	Cyperaceae	T	✓	South America	S		Naturalised
Cyrtomium falcatum (L. f.) C. Presl	Dryopteridaceae	H	✓	East Asia	W (M)	x	Naturalised
Cystopteris fragilis (L.) Bernh.	Cystopteridaceae	H			M		
Cytisus spp.	Fabaceae	NP	✓	Culton	P	x	Casual
Dactylis glomerata L.	Poaceae	H			S (P)		
Danae racemosa (L.) Moench	Asparagaceae	G	✓	East Asia	S	x	Casual
Daucus carota L.	Apiaceae	H			S		
Delosperma cooperi (Hook.f.) L. Bolus	Aizoaceae	CH		South Africa	P	x	
Dichondra micrantha Urb.	Convolvulaceae	G	✓	Asia	S (P)	x	Naturalised
Digitaria ischaemum Schreb. ex Muhl.	Poaceae	T			S		
Digitaria sanguinalis (L.) Scop.	Poaceae	T			S P		
Diospyros lotus L.	Ebenaceae	P	✓	East Asia	S	x	Naturalised
Diplotaxis muralis (L.) DC.	Brassicaceae	T			W		
Diplotaxis tenuifolia (L.) DC.	Brassicaceae	H			S (P) (W)	x	
Dipsacus fullonum L.	Dipsacaceae	H			S		
Dittrichia graveolens (L.) Greuter	Asteraceae	T			S P		
Dittrichia viscosa (L.) Greuter	Asteraceae	H			S P W		
Dryopteris filix-mas (L.) Schott	Dryopteridaceae	G			W M	x	
Dysphania pumilio (L.) Mosyakin & Clemants	Amaranthaceae	T	✓	Australia	P		Naturalised
Echinochloa crus-galli (L.) P. Beauv.	Poaceae	T			S (P)		
Eclipta prostrata (L.) L.	Asteraceae	T	✓	South America	S		Naturalised

Elaeagnus angustifolia L.	Elaeagnaceae	P	✓	Asia	S	x	Casual
Elaeagnus pungens Thunb.	Elaeagnaceae	P	✓	East Asia	S (P) (W)	x	Casual
Eleusine indica (L.) Gaertn.	Poaceae	T	✓	Asia	S P		Naturalised
Elymus repens (L.) Gould	Poaceae	G			S (P)		
Epilobium hirsutum L.	Onagraceae	H			S		
Epilobium montanum L.	Onagraceae	H			S		
Epilobium tetragonum L.	Onagraceae	H			P		
Equisetum arvense L.	Equisetaceae	G			S		
Equisetum ramosissimum Desf.	Equisetaceae	G			S		
Eragrostis barrelieri Daveau	Poaceae	T			P		
Eragrostis (cf.) *frankii* C.A. Mey. ex Steud.	Poaceae	T	✓	North America	P		Naturalised
Eragrostis mexicana subsp. *virescens* (J. Presl) S.D. Koch & Sánchez Vega	Poaceae	T	✓	South America	P		Naturalised
Eragrostis pectinacea (Michx.) Nees	Poaceae	T	✓	North America	P (S)		Naturalised
Eragrostis pilosa (L.) P. Beauv.	Poaceae	T			P (S)		
Erigeron annuus (L.) Desf.	Asteraceae	T	✓	North America	S P		Naturalised
Erigeron bonariensis L.	Asteraceae	T	✓	North America	S P (W)		Naturalised
Erigeron canadensis L.	Asteraceae	T	✓	North America	S P (W)		Naturalised
Erigeron karvinskianus DC.	Asteraceae	H	✓	South America	S P	x	Naturalised
Erigeron sumatrensis Retz.	Asteraceae	T	✓	South America	S P (W)		Naturalised
Eriobotrya japonica (Thunb.) Lindl.	Rosaceae	P	✓	East Asia	S	x	Casual
Erodium cicutarium (L.) L'Hér.	Geraniaceae	T			S (P)		
Erodium moschatum (L.) L'Hér.	Geraniaceae	T			S		
Erophila verna (L.) DC.	Brassicaceae	T			P W		
Euonymus europaeus L.	Celastraceae	P			S (W)		
Eupatorium cannabinum L.	Asteraceae	H			P		
Euphorbia amygdaloides L.	Euphorbiaceae	CH			S		
Euphorbia characias L.	Euphorbiaceae	NP			S	x	
Euphorbia helioscopia L.	Euphorbiaceae	T			S		
Euphorbia hirsuta L.	Euphorbiaceae	G			S		
Euphorbia lathyris L.	Euphorbiaceae	H	✓	West Europe	S P	x	Naturalised

Euphorbia maculata L.	Euphorbiaceae	T	✓	North America	S P		Naturalised
Euphorbia peplus L.	Euphorbiaceae	T			S (P)		
Euphorbia prostrata Aiton	Euphorbiaceae	T	✓	North America	S P		Naturalised
Fallopia convolvulus (L.) Á. Löve	Polygonaceae	T			S P		
Fallopia multiflora (Thunb.) Haraldson	Polygonaceae	P	✓	East Asia	S		Casual
Festuca rubra L.	Poaceae	H			S (P)	x	
Ficus carica L.	Moraceae	P			S P W (M)	x	
Foeniculum vulgare Mill.	Apiaceae	H			S P	x	
Fontanesia phillyraeoides Labill.	Oleaceae	P			S	x	
Fragaria vesca L.	Rosaceae	CH			S P		
Fraxinus excelsior L.	Oleaceae	P			P	x	
Fraxinus angustifolia subsp. *oxycarpa* (Willd.) Franco & Rocha Afonso	Oleaceae	P			S W (M)	x	
Fumaria capreolata L.	Papaveraceae	T			S		
Fumaria officinalis L.	Papaveraceae	T			S W		
Galanthus nivalis L.	Amaryllidaceae	G			S	x	
Galinsoga parviflora Cav.	Asteraceae	T	✓	South America	S		Naturalised
Galinsoga quadriradiata Ruiz & Pav.	Asteraceae	T	✓	South America	S		Naturalised
Galium album L.	Rubiaceae	H			S		
Galium aparine L.	Rubiaceae	T			S (P) (W) (R)		
Galium divaricatum Lam.	Rubiaceae	T			S P		
Galium murale (L.) All.	Rubiaceae	T			P		
Galium parisiense L.	Rubiaceae	T			P (W)		
Galium verum L.	Rubiaceae	T			S		
Geranium dissectum L.	Geraniaceae	T			S P		
Geranium molle L.	Geraniaceae	T			S P (R)		
Geranium nodosum L.	Geraniaceae	G			S	x	
Geranium purpureum Vill.	Geraniaceae	T			S P R		
Geranium rotundifolium L.	Geraniaceae	T			S (P)		
Geum urbanum L.	Rosaceae	H			S		
Ginkgo biloba L.	Ginkgoaceae	P	✓	East Asia	S	x	Casual

Glechoma hederacea L.	Lamiaceae	CH			S		
Gleditsia triacanthos L.	Fabaceae	P	✓	North America	S	x	Casual
Gnaphalium pensylvanicum Willd.	Asteraceae	H	✓	North America	P		Naturalised
Hedera algeriensis Hibbert	Araliaceae	P	✓	North Africa	S	x	Casual
Hedera helix L.	Araliaceae	P			S W (P)	x	
Hedysarum coronarium L.	Fabaceae	H			S		
Helianthus tuberosus L.	Asteraceae	G	✓	North America	S		Casual
Heliotropium amplexicaule Vahl	Boraginaceae	CH	✓	South America	S P		Naturalised
Heliotropium europaeum L.	Boraginaceae	T			S W		
Helleborus foetidus L.	Ranunculaceae	CH			S		
Helleborus viridis L.	Ranunculaceae	G			S		
Helminthotheca echioides (L.) Holub	Asteraceae	T			S (P)		
Hibiscus syriacus L.	Malvaceae	P	✓	Asia	S (M)	x	Casual
Hordeum murinum L.	Poaceae	T			S (P)		
Hordeum murinum subsp. *leporinum* (Link) Arcang.	Poaceae	T			S P (W) (R)		
Humulus lupulus L.	Cannabaceae	P			S		
Hyacinthoides hispanica (Mill.) Rothm.	Asparagaceae	G	✓	West Europe	S	x	Casual
Hyacinthus orientalis L.	Asparagaceae	G	✓	West Asia	S	x	Casual
Hylotelephium telephium (L.) H. Ohba	Crassulaceae	H	✓	Asia	S W	x	Casual
Hypericum perforatum L.	Hypericaceae	H			W (S)		
Hypochaeris radicata L.	Asteraceae	H			S P		
Iberis semperflorens L.	Brassicaceae	CH			W	x	
Ilex aquifolium L.	Aquifoliaceae	P			S	x	
Iris foetidissima L.	Iridaceae	G			S		
Iris x germanica L.	Iridaceae	G	✓	Culton	S (W)	x	Casual
Juglans nigra L.	Junglandaceae	P	✓	North America	S	x	Naturalised
Juglans regia L.	Junglandaceae	P	✓	Asia	S	x	Naturalised
Kalanchoe daigremontiana Raym.-Hamet & H. Perrier	Crassulaceae	CH		South Africa	P W	x	
Koelreuteria paniculata Laxm.	Sapindaceae	P	✓	Asia	S	x	

Laburnum anagyroides Medik.	Fabaceae	P			S	x	
Lactuca saligna L.	Asteraceae	T			S P (W) (R)	x	
Lactuca serriola L.	Asteraceae	H			S P W R		
Lamium amplexicaule L.	Lamiaceae	T			S (W)		
Lamium maculatum L.	Lamiaceae	H			S	x	
Lamium purpureum L.	Lamiaceae	T			S (W)		
Lapsana communis L.	Asteraceae	T			S (W)		
Lathyrus sylvestris L.	Fabaceae	H			S		
Laurus nobilis L.	Lauraceae	P			S	x	
Lepidium coronopus (L.) Al-Shehbaz	Brassicaceae	T			S		
Lepidium graminifolium L.	Brassicaceae	H			S		
Lepidium virginicum L.	Brassicaceae	T	✓	North America	S W		Naturalised
Leucanthemum maximum (Vaill.) Lam.	Asteraceae	H			S	x	
Ligustrum japonicum Thunb.	Oleaceae	P	✓	Asia	S	x	Casual
Ligustrum lucidum Aiton	Oleaceae	P	✓	Asia	S W (P)	x	Naturalised
Ligustrum sinense Lour.	Oleaceae	P	✓	Asia	S	x	Naturalised
Ligustrum vulgare L.	Oleaceae	NP			S	x	
Linaria (cf.) *vulgaris* Mill.	Scrophulariaceae	H			W		
Liquidambar styraciflua L.	Altingiacee	P	✓	North America	W	x	Casual
Lolium multiflorum Lam.	Poaceae	T			S		
Lolium perenne L.	Poaceae	H			S (P)	x	
Lonicera japonica Thunb.	Caprifoliaceae	P	✓	East Asia	S W	x	Naturalised
Lonicera ligustrina subsp. *yunnanensis* Franch.	Caprifoliaceae	P	✓	East Asia	S	x	Casual
Lotus corniculatus L.	Fabaceae	H			S		
Lotus tenuis Waldst. & Kit. ex Willd.	Fabaceae	H			S		
Lunaria annua L.	Brassicaceae	H			S		
Maclura pomifera (Raf.) C.K. Schneid.	Moraceae	P	✓	North America	S	x	Naturalised
Magnolia grandiflora L.	Magnoliaceae	P	✓	North America	S (W)	x	Naturalised
Mahonia aquifolium (Pursh) Nutt.	Berberidaceae	NP	✓	North America	S (W)	x	Naturalised
Mahonia japonica (Thunb.) DC.	Berberidaceae	NP	✓	Asia	S (W)	x	Naturalised
Malus domestica (Borkh.) Borkh.	Rosaceae	P	✓	Hybrid/Culton	S (W)		Naturalised

Malva sylvestris L.	Malvaceae	H			S (P)		
Medicago arabica (L.) Huds.	Fabaceae	T			S		
Medicago lupulina L.	Fabaceae	T			S		
Medicago minima (L.) L.	Fabaceae	T			W (S) (P)		
Medicago sativa L.	Fabaceae	T			S		
Melia azedarach L.	Meliaceae	P	✓	Asia	S P	x	Casual
Melica uniflora Retz.	Poaceae	H			S	x	
Melilotus officinalis (L.) Pall.	Fabaceae	H			S		
Mentha spicata L.	Lamiaceae	H			S	x	
Mercurialis annua L.	Euphorbiaceae	T			S (P)		
Mirabilis jalapa L.	Nyctaginaceae	G	✓	South America	S	x	Casual
Morus alba L.	Moraceae	P	✓	East Asia	S P W	x	Naturalised
Muscari comosum (L.) Mill.	Asparagaceae	G			S		
Muscari neglectum Guss. ex Ten.	Asparagaceae	G			S W		
Myosotis arvensis (L.) Hill	Boraginaceae	T			S		
Myosotis ramosissima Rochel ex Schult.	Boraginaceae	T			S		
Nandina domestica Thunb.	Berberidaceae	NP	✓	Asia	S	x	Casual
Narcissus ex-cv-group 2 Hort.	Amaryllidaceae	G	✓	Culton	S	x	Naturalised
Nerium oleander L.	Apocynaceae	P			P (S)	x	
Ocimum basilicum L.	Lamiaceae	H	✓	N.D.	P	x	Casual
Olea europaea L.	Oleaceae	CH			S	x	
Ophiopogon japonicus (Thunb.) Ker Gawl	Asparagaceae	G	✓	East Asia	S	x	Casual
Orchis purpurea Huds.	Orchidaceae	G			S		
Ornithogalum umbellatum L.	Asparagaceae	G			S		
Orobanche hederae Duby	Orobancaceae	T			S		
Ostrya carpinifolia Scop.	Betulaceae	P			S (P)	x	
Oxalis articulata Savigny	Oxalidaceae	G	✓	South America	S	x	Naturalised
Oxalis corniculata L.	Oxalidaceae	CH			S P W (M)		
Oxalis dillenii Jacq.	Oxalidaceae	H	✓	North America	S P		Naturalised
Oxalis pes-caprae L.	Oxalidaceae	G	✓	South Africa	S		Naturalised
Papaver dubium L.	Papaveraceae	T			S		

Papaver rhoeas L.	Papaveraceae	T			S W		
Parietaria judaica L.	Urticaceae	H			S W (S) (R)		
Parietaria officinalis L.	Urticaceae	H			S P W M		
Parthenocissus quinquefolia (L.) Planch.	Vitaceae	P	✓	North America	S	x	Naturalised
Passiflora coerulea L.	Passifloraceae	P	✓	South America	S P	x	Casual
Paulownia tomentosa (Thunb.) Steud.	Scrophulariaceae	P	✓	East Asia	S W	x	Casual
Petasites fragrans (Vill.) C. Presl	Asteraceae	G			S	x	
Petunia hybrida Vilm.	Solanaceae	T	✓	South America	S	x	Casual
Phalaris brachystachys Link	Poaceae	T			S		
Philadelphus coronarius L.	Hydrangeaceae	NP			S	x	
Phoenix canariensis Chabaud	Palmae	P	✓	North Africa	S	x	Casual
Phragmites australis (Cav.) Trin. ex Steud.	Poaceae	G			S	x	
Phytolacca americana L.	Phytolaccaceae	G	✓	North America	S		Naturalised
Picris hieracioides L.	Asteraceae	H			S P		
Pinus pinea L.	Pinaceae	P			W	x	
Pistacia terebinthus L.	Anacardiaceae	P			W		
Pisum sativum L.	Fabaceae	P	✓	Culton	P		Casual
Pittosporum tobira (Thunb.) W.T. Aiton	Pittosporaceae	P	✓	East Asia	S	x	Casual
Plantago coronopus L.	Plantaginaceae	T			P		
Plantago lanceolata L.	Plantaginaceae	H			S (P)		
Plantago major L.	Plantaginaceae	H			S P		
Platanus x hispanica Mill. ex Münchh.	Platanaceae	P	✓	Culton	P (W) (M)		Naturalised
Platycladus orientalis (L.) Franco	Cupressaceae	P	✓	East Asia	P	x	Casual
Poa annua L.	Poaceae	T			S P W R (M)		
Poa bulbosa L.	Poaceae	H			S W		
Poa pratensis L.	Poaceae	H			S (P)	x	
Poa trivialis L.	Poaceae	H			S (P)	x	
Polycarpon tetraphyllum (L.) L.	Caryophyllaceae	T			P (S)		
Polygonum aviculare L.	Polygonaceae	T			S P		
Polypodium interjectum L.	Polypodiaceae	H			R		
Polypogon viridis (Gouan) Breistr	Poaceae	H	✓	Africa			Naturalised

Poncirus trifoliata (L.) Raf.	Rutaceae	P	✓	East Asia	S	x	Casual
Populus alba L.	Salicaceae	P			S (P)	x	
Populus nigra L.	Salicaceae	P			P	x	
Portulaca oleracea L.	Portulacaceae	T			S P		
Potentilla indica (Jacks.) Th. Wolf	Rosaceae	H	✓	East Asia	S P		Naturalised
Potentilla reptans L.	Rosaceae	H			S P		
Primula vulgaris Huds.	Primulaceae	H			S		
Prunella vulgaris L.	Lamiaceae	H			S		
Prunus armeniaca L.	Rosaceae	P	✓	Asia	S		Casual
Prunus avium L.	Rosaceae	P			S W (P)		
Prunus cerasifera Ehrh.	Rosaceae	P	✓	West Asia	S (W)	x	Naturalised
Prunus cerasus L.	Rosaceae	P	✓	N.D.	S	x	Naturalised
Prunus dulcis (Mill.) D.A. Webb	Rosaceae	P	✓	Asia	S W		Casual
Prunus laurocerasus L.	Rosaceae	P	✓	West Asia	S	x	Casual
Prunus persica (L.) Batsch	Rosaceae	P	✓	Asia	S		Casual
Prunus pissardii Ehrh.	Rosaceae	P	✓	West Asia	S (W)	x	Naturalised
Prunus spinosa L.	Rosaceae	P			S	x	
Pteris multifida Poir.	Pteridaceae	G	✓	East Asia	M		Naturalised
Pteris vittata L.	Pteridaceae	H			W		
Pulmonaria officinalis L.	Boraginaceae	H			S		
Punica granatum L.	Lythraceae	P	✓	East Europe	S	x	Naturalised
Pyracantha rogersiana (A.B. Jacks.) Coltm.-Rog.	Rosaceae	P	✓	East Asia	S W	x	Naturalised
Pyrus communis L.	Rosaceae	P	✓	Culton	S		Casual
Quercus cerris L.	Fagaceae	P			S (W)	x	
Quercus ilex L.	Fagaceae	P			S W	x	
Quercus pubescens Willd.	Fagaceae	P			S W (P)	x	
Quercus robur L.	Fagaceae	P			S (W)	x	
Ranunculus repens L.	Ranunculaceae	H			S		
Ranunculus acris L.	Ranunculaceae	H			S		
Ranunculus bulbosus L.	Ranunculaceae	H			S		
Ranunculus ficaria L.	Ranunculaceae	G			S		

Ranunculus neapolitanus Ten.	Ranunculaceae	H			S (W)		
Ranunculus parviflorus L.	Ranunculaceae	T			S		
Ranunculus sardous Crantz	Ranunculaceae	T			S		
Robinia pseudoacacia L.	Fabaceae	P	✓	North America	S		Naturalised
Rorippa sylvestris (L.) Besser	Brassicaceae	H			S (P)		
Rosa canina L.	Rosaceae	NP			S		
Rosmarinus officinalis L.	Lamiaceae	NP			W	x	Casual
Rostraria cristata (L.) Tzvelev	Poaceae	T			P		
Rubus caesius L.	Rosaceae	NP			S		
Rubus ulmifolius Schott	Rosaceae	NP			S (W)		
Rumex crispus L.	Polygonaceae	H			S (P)		
Rumex pulcher L.	Polygonaceae	H			S		
Ruscus aculeatus L.	Asparagaceae	CH			S	x	
Sagina apetala Ard.	Caryophyllaceae	T			P (S)		
Sagina procumbens L.	Caryophyllaceae	H			P (S)		
Salix alba L.	Salicaceae	P			P		
Salix caprea L.	Salicaceae	P			P		
Salix cinerea L.	Salicaceae	P			P		
Salpichroa origanifolia (Lam.) Thell.	Solanaceae	CH	✓	South America	S		Naturalised
Salvia pratensis L.	Lamiaceae	H			S		
Salvia verbenaca L.	Lamiaceae	H			S		
Sambucus ebulus L.	Caprifoliaceae	G			S		
Sambucus nigra L.	Caprifoliaceae	P			S W		
Sanguisorba minor Scop.	Rosaceae	H			S		
Saxifraga stolonifera Curtis	Saxifragaceae	H	✓	East Asia	S	x	Casual
Saxifraga tridactylites L.	Saxifragaceae	T			P W R (M)		
Schedonorus pratensis (Huds.) P. Beauv.	Poaceae	H			S	x	
Scilla bifolia L.	Asparagaceae	G			S	x	
Sedum acre L.	Crassulaceae	CH			W R		
Sedum album L.	Crassulaceae	CH			P R		
Sedum dasyphyllum L.	Crassulaceae	CH			R		

Sedum hispanicum L.	Crassulaceae	T			W R		
Sedum lineare Thunb.	Crassulaceae	CH	✓	East Asia	W	x	Casual
Sedum palmeri S. Watson	Crassulaceae	CH	✓	South America	P W R M	x	Naturalised
Sedum pseudorupestre L.	Crassulaceae	CH			P R	x	
Senecio inaequidens DC.	Asteraceae	T	✓	South Africa	S P R		Naturalised
Senecio vulgaris L.	Asteraceae	T			S P W R		
Senecio spp.	Asteraceae	H	✓	Culton	S	x	Casual
Setaria pumila (Poir.) Roem. & Schult.	Poaceae	T			S P		
Setaria verticillata Dumort.	Poaceae	T			P		
Setaria viridis (L.) P. Beauv.	Poaceae	T			S P (W)		
Silene flos-cuculi (L.) Clairv.	Caryophyllaceae	H			S		
Silene latifolia subsp. *alba* (Mill.) Greuter & Burdet	Caryophyllaceae	H			S		
Sinapis arvensis L.	Brassicaceae	T			S R		
Sisymbrium irio L.	Brassicaceae	T			P (S)		
Sisymbrium officinale (L.) Scop.	Brassicaceae	T			S P		
Solanum pseudocapsicum L.	Solanaceae	CH	✓	South America	S	x	Naturalised
Solanum dulcamara L.	Solanaceae	NP			S		
Solanum lycopersicum L.	Solanaceae	T	✓	South America	S P		Naturalised
Solanum nigrum L.	Solanaceae	T			S P (R)		
Solanum villosum Mill.	Solanaceae	T			S		
Soleirolia soleirolii (Req.) Dandy	Urticaceae	H			S		
Sonchus asper (L.) Hill	Asteraceae	T			S P (W)		
Sonchus oleraceus L.	Asteraceae	T			T P (m) (s) (v)		
Sonchus tenerrimus L.	Asteraceae	T			S P (M)		
Sophora japonica L.	Fabaceae	P	✓	East Asia	S (P)	x	Naturalised
Sorghum halepense (L.) Pers.	Poaceae	G			S (P)		
Stachys annua (L.) L.	Lamiaceae	T			S		
Stellaria aquatica (L.) Scop.	Caryophyllaceae	H			S		
Stellaria media (L.) Vill.	Caryophyllaceae	T			S P W R M		
Sternbergia lutea (L.) Ker Gawl. ex Spreng.	Amaryllidaceae	G			S	x	

Symphoricarpos x chenaultii (L.) S.F. Blake	Caprifoliaceae	P	✓	North America	S	x	Casual
Symphyotrichum lanceolatum (Willd.) G.L. Nesom	Asteraceae	H	✓	North America	S P		Naturalised
Symphyotrichum squamatum (Spreng.) G.L. Nesom	Asteraceae	T	✓	South America	S P		Naturalised
Symphytum bulbosum K.F. Schimp.	Boraginaceae	G			S		
Symphytum officinale Lepech.	Boraginaceae	H			S	x	
Symphytum orientale L.	Boraginaceae	H	✓	Asia	S		Naturalised
Symphytum tuberosum L.	Boraginaceae	G			S (P)		
Syringa vulgaris L.	Oleaceae	P	✓	East Europe	S (W)	x	Casual
Taraxacum officinale L.	Asteraceae	H			S P M (W)		
Taxus baccata L.	Taxaceae	P			S W	x	
Thelypteris palustris Schott	Thelypteridaceae	G			S	x	
Thlaspi arvense L.	Brassicaceae	T			S		
Thymus (cf.) *serpyllum* L.	Lamiaceae	CH			W		
Tilia americana L.	Tiliaceae	P	✓	North America	S	x	Casual
Torilis arvensis (Huds.) Link	Apiaceae	T			S (M)		
Torilis nodosa (L.) Gaertn.	Apiaceae	T			S (P)		
Trachycarpus fortunei (Hook.) H. Wendl.	Palmae	P	✓	East Asia	S	x	Naturalised
Tribulus terrestris L.	Zygophyllaceae	T			S		
Trifolium campestre Schreb.	Fabaceae	T			S		
Trifolium fragiferum L.	Fabaceae	CH			S		
Trifolium pratense L.	Fabaceae	CH			S P		
Trifolium repens L.	Fabaceae	CH			S P (W)		
Trifolium scabrum L.	Fabaceae	T			W (P)		
Trisetaria flavescens (L.) Baumg.	Poaceae	H			S (W)		
Triticum aestivum L.	Poaceae	T	✓	Culton	S P		Naturalised
Tulipa gesneriana L.	Liliaceae	G	✓	East Europe	S	x	Casual.
Tussilago farfara L.	Asteraceae	G			S (P)		
Ulmus minor Mill.	Ulmaceae	P			S P (M)	x	
Ulmus pumila L.	Ulmaceae	P	✓	East Asia	S P (W)	x	Naturalised

Umbilicus rupestris (Salisb.) Dandy	Crassulaceae	G			W R (P)		
Urtica dioica L.	Urticaceae	H			S		
Urtica urens L.	Urticaceae	T			S		
Verbascum blattaria L.	Scrophulariaceae	H			S		
Verbascum (cf.) *phlomoides* L.	Scrophulariaceae	H			S		
Verbascum sinuatum L.	Scrophulariaceae	H			S (W)		
Verbena officinalis L.	Verbenaceae	H			S (P)		
Veronica arvensis L.	Plantaginaceae	T			S P		
Veronica chamaedrys L.	Plantaginaceae	H			S		
Veronica cymbalaria Bodard	Plantaginaceae	T			W		
Veronica hederifolia L.	Plantaginaceae	T			S P W		
Veronica peregrina L.	Plantaginaceae	T	✓	North America	P		Naturalised
Veronica persica Poir.	Plantaginaceae	T	✓	West Asia	S P (W)		Naturalised
Veronica polita Fr.	Plantaginaceae	T			W (S) (P)		
Viburnum tinus L.	Rosaceae	P			S	x	
Vicia sativa L.	Fabaceae	T			S W		
Vinca major L.	Apocynaceae	CH			S	x	
Vinca minor L.	Apocynaceae	CH			S	x	
Viola alba Besser	Violaceae	H			S		
Viola (cf.) *riviniana* Rchb.	Violaceae	H			S (W)		
Viola odorata L.	Violaceae	H			S P (W)		
Vitis labrusca L.	Vitaceae	P	✓	North America	S		Naturalised
Vitis vinifera L.	Vitaceae	P			S		
Vulpia myuros (L.) C.C. Gmel.	Poaceae	T			S P		
Wisteria sinensis (Sims) Sweet	Fabaceae	P	✓	East Asia	S	x	Casual
Unidentified genus and species	Fabaceae	T			P		
Unidentified genus and species	Apiaceae	H			S		
Unidentified genus and species	Asteraceae	H			S		
Unidentified genus and species	Asteraceae	NP	✓	N.D.	P		Casual
Unidentified genus and species	Brassicaceae	T	N.D.		S		
Unidentified genus and species	Fam. 1	T	N.D.		S		

Unidentified genus and species	Fam. 2	H	N.D.		S		

[1] Hetterscheid W.L.A., Brandenburg W.A. Culton versus taxon: Conceptual issues in cultivated plant systematics. *Taxon,* **44:** 161-175 (1995).

[2] Pignatti, S. *Flora d'Italia*. Bologna, Italy (Edagricole, 1982).

[3] Galasso, G. *et al.* An updated checklist of the vascular flora alien to Italy. *Plant Biosyst* **152**, 556-592 (2018).
